# Interocular Symmetry of Choroidal Parameters in Patients with Diabetic Retinopathy with and without Diabetic Macular Edema

**DOI:** 10.3390/jcm13010176

**Published:** 2023-12-28

**Authors:** Anna Raciborska, Patryk Sidorczuk, Joanna Konopińska, Diana Anna Dmuchowska

**Affiliations:** Ophthalmology Department, Medical University of Bialystok, 24a M. Sklodowskiej-Curie, 15-276 Bialystok, Poland; okulistyka@uskwb.pl (A.R.); joanna.konopinska@umb.edu.pl (J.K.)

**Keywords:** choroidal vascularity index, choroidal volume, choroidal thickness, diabetic macular edema, diabetic retinopathy, interocular symmetry

## Abstract

This study focuses on the interocular comparison of choroidal parameters in diabetic patients with diabetic retinopathy (DR) with and without diabetic macular edema (DME), as well as in patients with unilateral DME (present in only one eye). The aim of this study was to determine the symmetry in order to obtain better insights into the pathophysiology of diabetic choroidopathy. This retrospective single-center cross-sectional study included 170 eyes from 85 patients (61 with DR and 24 controls), divided into subgroups depending on the presence of DME. The patients underwent fluorescein angiography and spectral domain optical coherence tomography examination, and the analysis included various choroidal parameters: choroidal thickness, volume, and the choroidal vascularity index (CVI). In terms of the choroidal thickness, one eye of a patient with DR, regardless of the presence, absence, or unilaterality of DME, may be treated as representative for that patient. CVI proved symmetrical for controls and patients with DR without DME. However, there was some asymmetry of CVI in patients with bilateral or unilateral DME. There was no straightforward relationship between choroidopathy and DME. Other mechanisms were also involved in the pathogenesis.

## 1. Introduction

The human body is superficially anatomically symmetrical about the left–right axis, while the internal organs have a high degree of asymmetry [1]. Even paired organs such as the eyes are not equivalent [2]. Healthy patients show asymmetry in the parameters of the cornea, retina, eyeball length, and optic nerve [3,4,5,6,7]. Although generally symmetrical, various diseases develop unevenly in the eyes of a given patient, e.g., age-related macular degeneration (AMD) [8,9] and glaucoma [10]. There are also differences between the eyes in the central serous chorioretinopathy (CSCR) [11] and myopia [12,13].

Diabetic retinopathy is a common cause of blindness in adults in developed countries. Studies suggest that diabetic retinopathy usually develops symmetrically in both eyes of the patient [14,15], but it may also occur asymmetrically or even unilaterally. Laron et al. demonstrated the interocular symmetry of locations with abnormal multifocal electroretinogram (mfERG) between the right and left eyes in adolescents with type 1 diabetes and no DR [16]. Iino et al. reported that in their study, only 8.4% of patients demonstrated unilateral DR [17]. On the other hand, there are reports on unilateral DR [18,19]. Those authors suggest that in such cases additional pathological processes should be considered, e.g., unilateral carotid or ophthalmic artery disease. DME is usually bilateral but may occur unilaterally [20,21,22,23].

The relationships between DR, DME, and choroidopathy are complex. Diabetes affects both the retinal and choroidal circulation. DME can occur at any stage of DR. Moreover, choroidopathy is one of the components in the multifactorial pathogenesis of DR and DME [24,25,26]. However, the exact relationship remains unclear, and we were able to show that the involvement of the retinal and choroidal vasculature seems to occur independently from one another [27]. The choroid is highly vascularized and supplies one-third of the outer retina with oxygenated blood [28]. It can be characterized by parameters, such as choroid thickness (CT), volume (CV), and choroidal vascularity index (CVI). CVI is a relatively new indicator, calculated as the ratio of the luminal area (LA) relative to the total choroidal area (TCA). CVI is useful for assessing the state of choroidal vascularity, and unlike CT, it is not susceptible to the influence of various factors, which makes it a stable and reliable parameter [29]. The choroid between the left and right eye may differ due to the course of its vascularization and the anatomical structure of large vessels—the right common carotid artery departs from the brachiocephalic trunk, while the left one departs from the aorta [30]. On the other hand, the thickness of the choroid in healthy people between the eyes is a symmetrical parameter [31,32,33,34]. However, there are studies that indicate a difference between the thickness of the choroid in the nasal part of the macula in the right and left eyes [35]. Other studies point to good or moderate symmetry of CVI in healthy people [30,36]. Thus, the data regarding the symmetry of choroidal parameters in healthy subjects are inconclusive. On the metabolomic level, we were able to show a high degree of interocular symmetry of the aqueous humor composition in healthy subjects [37]. The choroidal parameters, such as the macular thickness, total area (TCA), stromal choroidal area (SCA), luminal area (LA), and the choroidal vascularity index (CVI) in the eye with greater myopia, are smaller than in the fellow eye in subjects with anisometropia. Therefore, the blood flow in the eye with such a thinned choroid is disturbed [38].

To the best of our knowledge, there are no studies that assess the symmetry of choroidal parameters between the eyes in patients with diabetic retinopathy with or without DME or in patients with unilateral DME. Knowledge on this subject could facilitate a better understanding of the pathophysiology of diabetes-related choroidopathy and the symmetry of the disease progression. This is important, among others, when planning subsequent clinical trials in order to decide whether a randomly selected eye included in the trial can be representative of a given patient. Moreover, bearing in mind that diabetic retinopathy progresses symmetrically between the right and left eyes in most cases [14], a comparative examination of both eyes may contribute to the early identification of complications or systemic diseases causing a unilateral form of DR [39].

## 2. Materials and Methods

The retrospective single-center cross-sectional study included 170 eyes from 85 patients (61 with DR and 24 controls). Medical records were analyzed for the timespan from 28 February 2017 to 20 February 2021. In the DR group, fluorescein angiography was performed with a Spectralis HRA+OCT imaging device (Heidelberg Engineering, Heidelberg, Germany) in a standard manner. The severity of DR was assessed according to the ETDRS criteria [40,41]. Depending on the presence of DME, the DR group was divided into three subgroups: DME present bilaterally (DR+DME+) or lack thereof (DR+DME−) or present in one eye and absent in the other eye (unilateral DME). The control group comprised patients undergoing routine ocular examination (including fundoscopy) at the Department of Ophthalmology, University Teaching Hospital of Bialystok who did not have diabetes (self-reported).

Spectral-domain (SD) OCT examination was performed in all patients. The cut-off thickness for the diagnosis of DME was ≥300 µm in the central macular subfield (1 mm in diameter) of the Early Treatment Diabetic Retinopathy Study (ETDRS) grid sector [42]. The exclusion criteria for all groups included a history of posterior segment surgery or intravitreal injections, macular laser photocoagulation, macular changes due to other ocular diseases, ametropia ≥ 3.0 diopters, known ocular or systemic pathology that might affect the choroidal vasculature, glaucoma, and insufficient quality of the OCT images.

The study was performed in accordance with the Declaration of Helsinki and approved by the Ethics Committee at the Medical University of Bialystok (approval number APK.002.216.2020). Written informed consent was obtained from all participants.

### 2.1. Optical Coherence Tomography Images Acquisition and Analysis

We have previously described the protocol of the study in detail as in [43]. After mydriasis, the OCT images were taken between 8 a.m. and 11 a.m. to eliminate the influence of the time of the day on the choroidal thickness. Two investigators (P.S. and D.A.D.), blinded to the clinical characteristics of the assessed eyes, independently evaluated the images. Any inconsistencies were resolved through consensus discussions.

**Figure 1:**
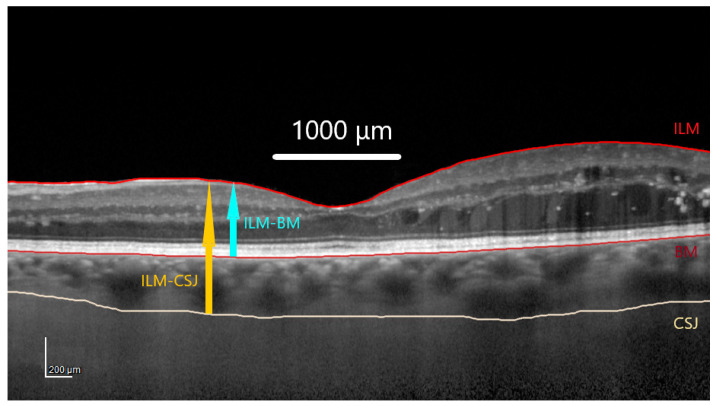
Marking of the choroidal–scleral junction, the sum of the choroidal and retinal thicknesses (ILM-CSJ), and retinal thickness (ILM-BM). Abbreviations: ILM, internal limiting membrane; BM, Bruch’s membrane; CSJ, choroidal–scleral junction.

According to the protocol, SD-OCT images were taken with a Spectralis HRA+OCT imaging device with eye-tracking (Heidelberg Engineering, Heidelberg, Germany). Twenty-five horizontal raster scans (20 × 20°) and a linear B-scan centered at the fovea were performed. The Bruch’s membrane (BM) line was shifted manually to the choroidal–scleral junction on each scan, as described previously in [43]. The authors reviewed the measurements, and disagreements were resolved through discussion. Retinal parameters obtained automatically from the ILM to the BM were then subtracted from the total parameters (obtained automatically from the ILM to the manually marked choroidal–scleral junction) (Figure 1) to get the values of the choroidal parameters. The fovea was checked and manually replotted whenever indicated. The choroid was defined as an area between the BM and the choroidal–scleral junction. The ETDRS macular map provided the values for the central 1 mm ring (500 µm radius from the fovea). The choroidal central macular thickness was calculated by subtraction, as described above.

### 2.2. Binarization of Subfoveal Choroidal Images

Binarization and segmentation of the images were conducted with ImageJ software (http://imagej.nih.gov/ij, 5 May 2021, version 1.49) according to the protocol proposed by Sonoda [44,45]. Briefly, the area of 500 µm nasally and temporally from the fovea was evaluated on the foveal horizontal scan using the polygon selection tool. The BM was determined as the upper border and the choroidal–scleral junction as the lower one. The luminal area (LA) and total choroidal area (TCA) were calculated [46]. The stromal choroidal area (SCA) was measured, and the CVI was calculated as the LA-to-TCA ratio [44,47]. The absolute agreement model of the intraclass correlation coefficient (ICC) reflected the inter-grader reliability. The ICC values for the choroidal parameters >0.8 showed good agreement. The fixed and proportional biases were excluded with Bland–Altman plot analyses.

### 2.3. Statistical Analysis

Analyses were conducted in the statistical software R, ver. 4.2.1. (R Core Team (2022). R: Language and environment for statistical computing by R Foundation for Statistical Computing, Vienna, Austria), with α = 0.05. The normality of the variables was analyzed with the Shapiro–Wilk’s test. Based on the result, the nonparametric Kruskal–Wallis test was chosen to compare quantitative variables between three groups (the Tukey post hoc test was conducted with Bonferroni corrections). Dependencies between groups and qualitative variables were assessed using the chi-square test. The analysis of the correlation between the left and right eyes was based on the ICC (intraclass correlation coefficient), with a 95% confidence interval (CI) as a primary analysis [48]. Additionally, as a secondary analysis, the relative mean difference between both eyes, the Spearman’s correlation coefficient (r) between both eyes, as well as the paired Wilcoxon test between both eyes (with Benjamini–Hochberg adjustments for multiple comparisons and without adjustment) were added.

According to the literature [49], ICC values < 0.5 are indicative of poor reliability, between 0.5 and 0.75 indicate moderate reliability, between 0.75 and 0.9 indicate good reliability, and values > 0.9 indicate excellent reliability. The relationship (or the correlation) between the two variables is quantified with a number (range −1 and +1). Zero indicates no correlation, and 1 reflects a complete or perfect correlation. The interpretation of Pearson’s and Spearman’s correlation coefficients (r) according to Chan YH is presented in Table 1 [50].

## 3. Results

### 3.1. Baseline Characteristics

Table 2 presents the demographic and clinical characteristics of the study groups. There were no significant differences in terms of age, sex, DR severity, and PRP distribution. The spherical equivalent was significantly lower among subjects from the DR−DME− group than the control group (*p* = 0.001 for main analysis, *p* = 0.002 for post hoc analysis) and the unilateral DME group (*p* < 0.001 for post hoc analysis) (Table 2).

### 3.2. Between-Group Comparison of Choroidal Parameters

Table 3 presents the values of the central macular choroidal thickness (µm), subfoveal choroidal thickness (SFCT), and the central macular and total choroidal volume, CVI, LA, SCA, and TCA. In general, the choroidal thickness parameters showed interocular symmetry in all groups. However, there were discrepancies regarding the CVI and SCA depending on the studied group (Table 4).

In all groups, the intraclass correlation coefficient (ICC) (i.e., how the data from one eye coincide with the data from the other eye) for selected choroidal parameters ranged from moderate to excellent, except for the poor reliability for CVI in the unilateral DME group ICC = 0.478 CI_95_ [0.000; 0.775] and the SCA in the controls ICC = 0.373 CI_95_ [−0.029; 0.671].

Spearman’s correlation coefficient (r) showed a strong correlation for most of the parameters, except the CVI in the unilateral DME group, where the correlation was statistically not significant (r = 0.333, *p* = 0.191), as well as the SCA in controls (r = 0.331, *p* = 0.114).

The Wilcoxon test, determining whether the average level for the left eye differs significantly compared to the right eye, was only significant for the CVI in the DR+DME− group (*p* = 0.024). However, there was a good correlation between the right and left eyes (ICC = 0.774 CI_95_ [0.489; 0.893] and r = 0.850, *p* < 0.001).

Furthermore, although the average levels of the CVI between the eyes in DR+DME+ did not differ significantly (*p* = 0.919), the correlation was rather poor (fair r, moderate ICC).

## 4. Discussion

In this study, we compared choroidal parameters between eyes in patients with diabetic retinopathy with or without DME or with unilateral DME in comparison with controls. Our study demonstrated that choroidal thickness proved symmetrical for all studied groups. However, there was some asymmetry of the CVI in patients with bilateral or unilateral DME. Understanding interocular symmetry could be an important factor in the diagnosis, treatment, and follow-up of various diseases [31]. The studied groups showed no differences in terms of sex, age, the severity of DR, or the presence of panphotocoagulation (PRP). However, differences in the spherical equivalent were observed, which is most often related to the length of the eyeball. According to Chen et al. [32], the length of the eyeball (AXL) has a significant impact on the subfoveal choroidal thickness (SFCT), showing a strong negative correlation, while according to Iovino et al. [51], the AXL does not have a significant effect on the CVI. In order to exclude this potential confounding factor, ametropia ≥ 3 diopters was used as an exclusion criterion.

There are discrepancies in the literature regarding the CT in eyes with DME. Some authors describe a decrease in CT in connection with DR and DME [52,53,54]. Some studies report an increase in CT in the course of DME [55,56], whereas others show no relationship between CT and DME [57,58]. These discrepancies may be caused by a multitude of factors influencing the parameters of the choroid, especially its thickness. The CT, as opposed to CVI, depends on sex, age, and eyeball length, according to research conducted by Agrawal et al. [29]. Studies describe a reduction in the CVI in the eyes of diabetics compared to healthy people [55,59], which is also confirmed in our study.

Regarding the CT, we found no interocular differences. However, we found discrepancies regarding CVI depending on the studied group. The asymmetry of the CVI was demonstrated in the unilateral DME group. Furthermore, in the DR+DME+ group, although the average levels of the CVI between the eyes did not differ significantly, the correlation was rather poor (fair r, moderate ICC). In summary, the CVI from one eye seems to be representative of the fellow eye in the controls and the DR+DME− group but not in the unilateral DME and DR+DME+ groups.

This may be due to the fact that choroidopathy is only one of the components of the DME pathogenesis. DME has a more complex pathomechanism, in which ischemia, neurodegeneration, swelling, vascular endothelial growth factor (VEGF), and disorder of the blood–retinal barrier play a role [60]. In addition, diabetes leads to vascular disorders of the choroid and retina, but these changes occur independently of each other [27]. It is worth adding that DME can occur at every stage of the development of diabetic retinopathy and is not related to its severity [61].

The pathogenetic factors for DME are on the systemic and local levels. Thanks to the inclusion of both eyes of the same patients, the systemic factors seem to be to some extent negligible as they affect both eyes. This may explain the symmetry of the process taking place in the eyes of a patient suffering from diabetes. However, when the multifactorial and complex nature of the pathophysiology of DME is considered, the choroidal asymmetries may be more evident in more advanced cases.

Our study has its limitations. The study was retrospective. We did not have access to the data such as the duration of the disease, blood pressure, and glycated hemoglobin, but according to Agrawal et al., these variables do not affect the CVI [47]. The single-center setting of the study could limit the generalizability of the results. Fluorescein angiography was performed. The assessment of DR severity was based on ETDRS criteria. Information regarding more peripheral retinal changes obtained with widefield or ultra-widefield fluorescein angiography could be of additional value.

This study also has the following strengths. We are the first to address the subject of interocular symmetry of the choroidal parameters in patients with diabetes. The inclusion of patients with unilateral DME is unique. The subject of symmetry, which has so far been rarely analyzed in research, is innovative. The CVI enabled detailed characterization of the choroid. The volume of the choroid is a parameter quite rarely evaluated and shows an advantage over the assessment of the thickness of the choroid because it takes into account the irregularity of the shape of the choroid, unlike CT, which is measured on a single scan [62]. By performing OCT tests at a similar time of day, we avoided the impact of this factor on the results.

In future studies, a three-dimensional automated CVI algorithm would be beneficial. An interesting direction of research would be to include the results of a Doppler ultrasound of the internal carotid artery (ICA) and retrobulbar vessels.

## 5. Conclusions

Regarding pathogenesis, there is no straightforward relationship between choroidopathy and DME. Other mechanisms are also involved. In terms of CT, one eye in a patient with DR, regardless of the presence, absence, or unilaterality of DME, may be treated as representative for that patient. However, an assumption of the interocular symmetry of the CVI should be treated with greater caution, as there is some asymmetry of the CVI in patients with DR and bilateral or unilateral DME. These conclusions may facilitate the design of future studies and their proper interpretation.

## Figures and Tables

**Table 1:** Interpretation of Spearman’s and Pearson’s correlation coefficients (r) according to Chan YH [50].

Correlation Coefficient (r)		Interpretation
+1	−1	Perfect
+0.9	−0.9	Very strong
+0.8	−0.8	Very strong
+0.7	−0.7	Moderate
+0.6	−0.6	Moderate
+0.5	−0.5	Fair
+0.4	−0.4	Fair
+0.3	−0.3	Fair
+0.2	−0.2	Poor
+0.1	−0.1	Poor
0	0	None

**Table 2:** Baseline characteristics of patients with DR with or without concomitant DME or with unilateral DME (present in only one eye) and healthy controls.

Variables	Overall	Group				*p*
		DR+DME−	DR+DME+	Unilateral DME	Controls	
N of patients	102	40	21	17	24	-
N of eyes	204	80	42	34	48	-
Sex, male (%)	49 (48.0)	22 (55.0)	9 (42.9)	9 (52.9)	9 (37.5)	0.525
Age, years, median (Q1; Q3)	62.50 (52.00; 68.00)	59.00 (51.00; 68.00)	64.50 (55.50; 67.50)	69.00 (61.00; 71.00)	58.50 (45.50; 67.25)	0.113
Spherical equivalent, median (Q1; Q3)	0.15 (−0.09; 0.85)	0.06 (−0.25; 0.30) ^A^	0.02 (−0.12; 1.10)	0.44 (0.00; 1.51) ^B^	0.44 (−0.04; 1.13) ^B^	**0.001**
DR severity, n (%)						
NPDR	115 (73.7)	55 (68.8)	30 (71.4)	30 (88.2)	-	0.089
PDR	41 (26.3)	25 (31.2)	12 (28.6)	4 (11.8)	-	
PRP, n (%)						
No	115 (73.7)	59 (73.8)	30 (71.4)	26 (76.5)	-	0.884
Yes	41 (26.3)	21 (26.3)	12 (28.6)	8 (23.5)	-	

Abbreviations: DME, diabetic macular edema; DR, diabetic retinopathy; NPDR, nonproliferative diabetic retinopathy; PDR, proliferative diabetic retinopathy; PRP, panphotocoagulation; Q1, quartile 1; Q3, quartile 3. Dependencies between qualitative variables were analyzed using the chi-square test. Quantitative variables were compared using the Kruskal–Wallis test. Values with index ^A^ were lower than values with index ^B^ (in the Tukey post hoc test). *p* < 0.05 was considered statistically significant (highlighted with bold).

**Table 3:** Values of the choroidal parameters in fellow eyes.

Group	Variable	Mean ± SD Left Eyes	Mean ± SD Right Eyes	Median (Q1; Q3) Left Eyes	Median (Q1; Q3) Right Eyes	Relative Mean Difference (%) between Left and Right Eyes
DR+DME−	Central macular choroidal thickness (µm)	273.35 ± 58.13	269.75 ± 65.17	287.00 (239.25; 306.50)	277.00 (224.75; 321.75)	−4.49
	SFCT (µm)	274.62 ± 59.20	267.62 ± 68.57	285.00 (252.00; 307.00)	267.00 (221.25; 317.00)	−5.60
	Central macular choroidal volume (mm^3^)	0.22 ± 0.05	0.21 ± 0.05	0.22 (0.19; 0.24)	0.22 (0.18; 0.25)	−4.65
	Total choroidal volume (mm^3^)	7.40 ± 1.67	7.38 ± 1.64	7.61 (6.21; 8.36)	7.63 (6.22; 8.30)	−3.17
	CVI	0.62 ± 0.05	0.60 ± 0.06	0.62 (0.58; 0.66)	0.60 (0.55; 0.64)	−3.33
	LA (mm^2^)	1.42 ± 0.43	1.38 ± 0.43	1.47(1.11; 1.67)	1.42 (1.05; 1.67)	−5.92
	SCA (mm^2^)	0.86 ± 0.21	0.92 ± 0.24	0.86 (0.75; 1.02)	0.91 (0.76; 1.1)	1.72
	TCA (mm^2^)	2.29 ± 0.59	2.30 ± 0.61	2.38 (1.86; 2.69)	2.32 (1.85; 2.64)	−2.91
DR+DME+	Central macular choroidal thickness (µm)	244.76 ± 51.33	250.43 ± 43.66	238.00 (220.00; 274.00)	259.00 (220.00; 276.00)	2.29
	SFCT (µm)	247.05 ± 54.99	252.14 ± 51.15	245.00 (214.00; 274.00)	260.00 (219.00; 283.00)	2.04
	Central macular choroidal volume (mm^3^)	0.19 ± 0.04	0.20 ± 0.03	0.18 (0.18; 0.21)	0.20 (0.18; 0.22)	5.13
	Total choroidal volume (mm^3^)	6.66 ± 1.39	6.80 ± 1.13	6.62 (6.09; 7.41)	6.78 (6.08; 7.57)	2.08
	CVI	0.58 ± 0.05	0.57 ± 0.05	0.60 (0.52; 0.61)	0.57 (0.55; 0.61)	−1.74
	LA (mm^2^)	1.29 ± 0.39	1.29 ± 0.33	1.31 (1.03; 1.45)	1.28 (1.05; 1.6)	0.46
	SCA (mm^2^)	0.93 ± 0.22	0.95 ± 0.18	0.91 (0.76; 1.05)	0.91 (0.83; 1.09)	2.51
	TCA (mm^2^)	2.21 ± 0.57	2.24 ± 0.46	2.2 (1.9; 2.50)	2.31 (1.87; 2.64)	1.32
Unilateral DME *	Central macular choroidal thickness (µm)	* eye with DME 258.53 ± 48.95	* eye without DME 240.88 ± 59.83	255.00 (244.00; 294.00)	251.00 (185.00; 292.00)	7.07
	SFCT (µm)	262.71 ± 51.52	242.06 ± 59.95	263.00 (230.00; 301.00)	261.00 (190.00; 292.00)	8.18
	Central macular choroidal volume (mm^3^)	0.20 ± 0.04	0.19 ± 0.05	0.20 (0.19; 0.23)	0.20 (0.15; 0.23)	5.41
	Total choroidal volume (mm^3^)	6.70 ± 1.45	6.49 ± 1.51	6.78 (6.00; 7.54)	6.67 (5.27; 7.32)	3.23
	CVI	0.59 ± 0.05	0.58 ± 0.05	0.60 (0.55; 0.63)	0.59 (0.56; 0.62)	0.91
	LA (mm^2^)	1.28 ± 0.31	1.31 ± 0.34	1.23 (1.09; 1.41)	1.3(1.15; 1.46)	−1.61
	SCA (mm^2^)	0.91 ± 0.24	0.94 ± 0.26	0.99 (0.77; 1.12)	0.89 (0.81; 1.14)	−3.69
	TCA (mm^2^)	2.19 ± 0.51	2.25 ± 5.48	2.17 (1.80; 2.4)	2.22 (2.01; 2.53)	−2.47
Controls	Central macular choroidal thickness (µm)	306.54 ± 70.51	309.08 ± 67.91	302.50 (268.00; 348.50)	310.50 (244.50; 359.00)	0.83
	SFCT (µm)	304.67 ± 72.33	305.38 ± 69.62	297.00 (264.50; 332.75)	303.50 (247.50; 362.75)	0.23
	Central macular choroidal volume (mm^3^)	0.24 ± 0.06	0.24 ± 0.05	0.24 (0.21; 0.28)	0.24 (0.19; 0.28)	0.00
	Total choroidal volume (mm^3^)	8.33 ± 1.92	8.56 ± 1.92	8.25 (7.09; 9.71)	8.83 (6.96; 10.41)	2.72
	CVI	0.63 ± 0.06	0.63 ± 0.05	0.64 (0.59; 0.68)	0.63 (0.60; 0.68)	0.00
	LA (mm^2^)	1.58 ± 0.36	1.63 ± 0.39	1.63 (1.32; 1.81)	1.67 (1.34; 1.81)	2.99
	SCA (mm^2^)	0.9 ± 0.17	0.93 ± 0.184	0.88 (0.79; 0.99)	0.87 (0.79; 1.05)	3.33
	TCA (mm^2^)	2.48 ± 0.45	2.56 ± 0.52	2.53 (2.35; 2.75)	2.56 (2.26; 2.82)	3.11

Abbreviations: DME, diabetic macular edema; DR, diabetic retinopathy; SFCT, subfoveal choroidal thickness; CVI, choroidal vascularity index; LA, luminal area; SCA, stromal choroidal area; TCA, total choroidal area; Q1, quartile 1; Q3, quartile 3. * In the unilateral DME group, comparisons apply to DME+ eye vs. DME− eye.

**Table 4:** Comparison of choroidal parameters in fellow eyes.

	Variable	ICC between Left and Right Eyes	95% CI for ICC	r between Left and Right Eyes	*p* Value for r	*p*-Value of Wilcoxon Test (w/o Correction)	*p*-Value of Wilcoxon Test (with Correction)
DR+DME−	Central macular choroidal thickness	0.803	0.657; 0.890	0.758	**<0.001**	0.391	0.919
	SFCT	0.705	0.509; 0.832	0.634	**<0.001**	0.216	0.919
	Central macular choroidal volume	0.802	0.656; 0.890	0.768	**<0.001**	0.488	0.919
	Total choroidal volume	0.906	0.827; 0.950	0.855	**<0.001**	0.255	0.919
	CVI	0.774	0.489; 0.893	0.850	**<0.001**	0.001	0.024
	LA	0.856	0.745; 0.921	0.859	**<0.001**	0.289	0.919
	SCA	0.604	0.367; 0.768	0.612	**<0.001**	0.079	0.919
	TCA	0.781	0.622; 0.878	0.739	**<0.001**	0.868	0.919
DR+DME+	Central macular choroidal thickness	0.842	0.656; 0.932	0.859	**<0.001**	0.244	0.919
	SFCT	0.817	0.605; 0.921	0.745	**<0.001**	0.677	0.919
	Central macular choroidal volume	0.793	0.562; 0.910	0.796	**<0.001**	0.267	0.919
	Total choroidal volume	0.880	0.727; 0.950	0.923	**<0.001**	0.349	0.919
	CVI	0.522	0.119; 0.775	0.447	**0.044**	0.919	0.919
	LA	0.691	0.373; 0.862	0.714	**<0.001**	0.708	0.919
	SCA	0.529	0.133; 0.779	0.490	**0.026**	0.633	0.919
	TCA	0.674	0.347; 0.854	0.738	**<0.001**	0.838	0.919
Unilateral DME *	Central macular choroidal thickness	0.629	0.247; 0.846	0.639	**0.007**	0.187	0.374
	SFCT	0.518	0.095; 0.790	0.539	**0.028**	0.170	0.374
	Central macular choroidal volume	0.621	0.232; 0.842	0.582	**0.014**	0.345	0.513
	Total choroidal volume	0.868	0.680; 0.950	0.787	**<0.001**	0.449	0.513
	CVI	0.478	0.000; 0.775	0.333	0.191	0.963	0.963
	LA	0.727	0.387; 0.892	0.804	**<0.001**	0.098	0.374
	SCA	0.837	0.614; 0.937	0.850	**<0.001**	0.404	0.513
	TCA	0.849	0.638; 0.942	0.860	**<0.001**	0.120	0.374
Controls	Central macular choroidal thickness	0.802	0.593; 0.909	0.759	**<0.001**	0.864	0.919
	SFCT	0.761	0.519; 0.889	0.765	**<0.001**	0.891	0.919
	Central macular choroidal volume	0.818	0.623; 0.917	0.800	**<0.001**	0.881	0.919
	Total choroidal volume	0.854	0.696; 0.934	0.855	**<0.001**	0.456	0.919
	CVI	0.724	0.457; 0.871	0.775	**<0.001**	0.565	0.919
	LA	0.748	0.504; 0.882	0.798	**<0.001**	0.509	0.919
	SCA	0.373	−0.029; 0.671	0.331	0.114	0.790	0.919
	TCA	0.619	0.299; 0.815	0.732	**<0.001**	0.663	0.919

Abbreviations: DME, diabetic macular edema; DR, diabetic retinopathy; SFCT, subfoveal choroidal thickness; CVI, choroidal vascularity index; LA, luminal area; SCA, stromal choroidal area; TCA, total choroidal area; ICC, intraclass correlation coefficient; CI, confidence interval. * In the unilateral DME group, comparisons apply to DME+ eye vs. DME− eye. *p* < 0.05 was considered statistically significant (highlighted with bold).

## Data Availability

All the materials and information will be available upon an e-mail request to the corresponding author. Names and exact data of the participants of the study may not be available owing to patient confidentiality and privacy policy.
